# Fighting the Fire: Mechanisms of Inflammatory Gene Regulation by the Glucocorticoid Receptor

**DOI:** 10.3389/fimmu.2019.01859

**Published:** 2019-08-07

**Authors:** Laura Escoter-Torres, Giorgio Caratti, Aikaterini Mechtidou, Jan Tuckermann, Nina Henriette Uhlenhaut, Sabine Vettorazzi

**Affiliations:** ^1^Molecular Endocrinology, Helmholtz Zentrum München (HMGU), German Center for Diabetes Research (DZD), Institute for Diabetes and Cancer IDC, Munich, Germany; ^2^Department of Biology, Institute for Comparative Molecular Endocrinology, University of Ulm, Ulm, Germany; ^3^Gene Center, Ludwig-Maximilians-Universität (LMU), Munich, Germany

**Keywords:** glucocorticoid receptor, inflammation, macrophages, mouse models, gene regulation

## Abstract

For many decades, glucocorticoids have been widely used as the gold standard treatment for inflammatory conditions. Unfortunately, their clinical use is limited by severe adverse effects such as insulin resistance, cardiometabolic diseases, muscle and skin atrophies, osteoporosis, and depression. Glucocorticoids exert their effects by binding to the Glucocorticoid Receptor (GR), a ligand-activated transcription factor which both positively, and negatively regulates gene expression. Extensive research during the past several years has uncovered novel mechanisms by which the GR activates and represses its target genes. Genome-wide studies and mouse models have provided valuable insight into the molecular mechanisms of inflammatory gene regulation by GR. This review focusses on newly identified target genes and GR co-regulators that are important for its anti-inflammatory effects in innate immune cells, as well as mutations within the GR itself that shed light on its transcriptional activity. This research progress will hopefully serve as the basis for the development of safer immune suppressants with reduced side effect profiles.

## Introduction

### Glucocorticoids as Immunomodulators

Glucocorticoids (GCs) are steroid hormones secreted in a diurnal and stress responsive manner, under the control of the hypothalamic-pituitary-adrenal (HPA) axis (1).GCs regulate numerous essential physiological and developmental processes, ranging from lung maturation to glucose metabolism and immune responses. This is clearly demonstrated in mice with abrogated GC signaling, which die perinatally due to pulmonary atelectasis (2). The effect on lung maturation is not merely limited to mice: in clinical practice, pre-term neonates are given GCs to accelerate pulmonary development (3). In adult mammals, endogenous GCs play important homeostatic roles. For instance, GCs increase glucose production through glycogenolysis and gluconeogenesis in the liver upon fasting, and as part of daily rhythmic energy mobilization (4, 5).

Pharmacologically, GCs are widely used to treat acute and chronic inflammatory diseases, such as asthma, allergies, rheumatoid arthritis, inflammatory bowel disease, multiple sclerosis etc., due to their potent anti-inflammatory actions. In addition, GCs are commonly prescribed to prevent graft-vs.-host immune responses after organ transplantation and for certain cancer types, such as lymphoma (6, 7). Currently, it is estimated that 1–3% of the adult Western population are receiving GCs, demonstrating their broad applications (8). GCs have been used for over 70 years as anti-inflammatory drugs, despite their adverse effects on systemic metabolism, which were noted soon after their first clinical use (9). Long term exposure to GCs induces adipocyte hypertrophy, glucose intolerance and insulin resistance, hypertension, muscle and skin atrophy, osteoporosis, glaucoma, impaired wound healing and psychological effects such as mood changes, insomnia, and depression (4, 10). Long term GC exposure due to increased secretion from endocrine tumors or chronic exogenous administration, often causes a pathological condition known as Cushing's syndrome (11). Cushing's manifests as debilitating muscle wasting, fat accumulation, and susceptibility to infection and can be fatal if left untreated.

Separating beneficial therapeutic properties from detrimental side effects based on a molecular understanding of GC action is a long-term goal of biomedical research. Furthermore, the glucocorticoid receptor (GR) has been key to understanding the basic molecular concepts of GC action. There have been several paradigm shifts of the molecular understanding of GC/GR mechanisms since cloning of the receptor more than 30 years ago (12). The generation of GR mutants that interfere with specific functions of the receptor, the introduction of several mutants into preclinical models and the characterization of genome wide profiles all revolutionized our view of GC action. In this review, we summarize recent insights into the anti-inflammatory effects of GR, focusing on mechanisms of macrophage gene regulation, GR co-regulators, novel GR target genes, and mouse models of inflammation. We also summarize the current understanding of immune modulatory mechanism in the innate immune system based on mouse mutants. These might explain why, despite much progress, developing novel immune modulators that match the efficacy of GCs but avoid the adverse effects remains a major challenge for the field.

### The Glucocorticoid Receptor

**Figure 1:**
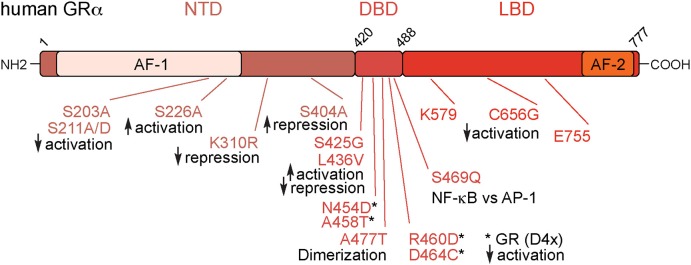
Overview of the glucocorticoid receptor protein. The Glucocorticoid Receptor (GR) is organized into three main domains: the N-terminal Transactivation Domain (NTD), the DNA-Binding Domain (DBD), and the Ligand Binding Domain (LBD). In addition, there are the transactivation domains 1 and 2 (AF-1 and AF-2). These mutations numbered above are relevant for GR's immunomodulatory effects. Numbers are amino acids of the human protein.

The endogenous GC, cortisol in humans and corticosterone in rodents, binds to the GR, encoded by the *NR3C1* gene. GR belongs to the nuclear receptor superfamily of ligand activated transcription factors. It consists of three major domains, the central DNA binding domain (DBD), the N-terminal transactivation domain (NTD), and the C-terminal ligand binding domain (LBD) [(12); Figure 1].

The *NR3C1* gene encodes several isoforms that are generated by alternative splicing and alternative initiation of translation (10, 13). The full-length isoform GRα-A is the focus of this review. GRβ, a second splice variant, and other GR isoforms, are known to modify GC sensitivity, but are discussed in detail elsewhere (14).

In the absence of ligand, GR resides in the cytoplasm, bound to heat shock proteins 70 and 90 (Hsp70 and Hsp90) together with other chaperones and immunophilins (15). Upon binding of GCs, GR translocates to the nucleus where it binds to DNA sequences. In this way, GR is recruited to target gene enhancers and promoters where it can both activate and repress transcription (16, 17). Canonical binding sites for the GR are called glucocorticoid response elements (GREs) and are composed of two 6bp palindromes (half sites) separated by a 3bp spacer, with the consensus AGAACAnnnTGTTCT. However, GR binding sites (GBS) in the genome vary to a certain degree of motif mismatch, expanding the number of possible target sequences. Furthermore, the context of neighboring transcription factor binding sites and the ensuing crosstalk is relevant for the regulation of inflammatory genes by the GR. The beauty of using GR as a model transcription factor is that its ability to regulate genes can be easily controlled *in vitro* and *in vivo* by the absence or presence of the GC ligand.

### Chromatin Residence Time and Multimerization of the Glucocorticoid Receptor

GR, along with other transcription factors, was assumed to bind DNA in a relatively static manner, “sitting down” for long periods of time to regulate gene expression. However, visualization of the dynamics of fluorescent-tagged GR in living cells led to the insight that occupancy of dimeric GR molecules at GREs is rather in the order of seconds and less (18). Only a small portion of available molecules are specifically bound to chromatin at a given time, suggesting that transcription factors and co-factors have a transient rather than stable interaction at genomic response elements (19).

GR acts as a monomer (20), dimer (21, 22), and even tetramer (23–25) depending on the subcellular localization, presence of ligand, GREs, or artificial response elements such as the MMTV array. Interestingly, DNA binding was proposed to trigger allosteric regulation of GR, followed by a change in its oligomeric state (24). Ligand bound GR is mainly nuclear and dimeric. Interestingly, upon DNA binding, the structural LBD rearrangement promotes the formation of higher order oligomers, predominantly tetramers, through unstudied LBD surfaces (25). The physiological relevance and implications of a tetrameric GR, however, are still open for debate and further investigation.

In general, chromatin binding and gene regulation by GR appear to be much more dynamic than previously thought, and the residence time of GR on chromatin may have differential effects. The LBD seems to regulate the number of GR molecules bound at a specific genomic region, which may also affect the transcription of target genes.

### Glucocorticoid Receptor Co-regulators

All nuclear receptors (NRs), including GR, require a host of co-activators and co-repressors to ultimately control the transcriptional apparatus.

**Figure 2:**
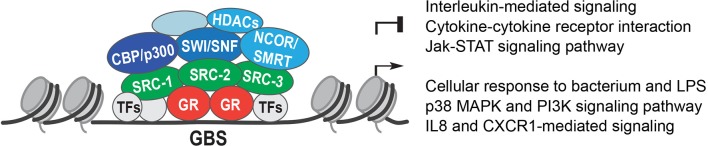
Glucocorticoid receptor co-regulators. The Glucocorticoid Receptor (GR) binds to Glucocorticoid Receptor Binding Sites (GBS) in open chromatin. GR interacts with other transcription factors (TFs) and recruits co-activators or co-repressors, such as: the Steroid Receptor co-activators 1, 2, and 3 (SRC-1, SRC-2, and SRC-3); the histone acetyl transferases CREB binding protein (CBP) and p300; the Nuclear Receptor co-repressors NCOR1 and NCOR2 (NCOR, SMRT), which recruit histone deacetylases 1 and 3 (HDACs); and the SWItch/Sucrose-Non Fermentable (SWI/SNF) chromatin remodeling complex.

Steroid receptor coactivator-1 (SRC-1, also known as nuclear receptor co-activator 1, NCOA1) was one of the first identified (26), followed by glucocorticoid receptor interacting protein (GRIP1, SRC-2, and NCOA2) (27). Originally found to be a co-activator of the progesterone receptor (PR), SRC-1, and GRIP1 were shown to directly interact with GR and other steroid receptors. This direct co-activator interaction with GR depends on the evolutionarily conserved LXXLL motif, or NR-box, and without this motif, GR loses transcriptional activity (28). SRC-1 directly activates genes with its histone acetyltransferase (HAT) domain that decondenses chromatin [(29); Figure 2].

The strength of GR's interaction with SRC-1 and GRIP1 might determine the steroid responsiveness of cancer cells, suggesting that the loss of GC-induced apoptosis or growth arrest is due to, at least in part, co-activator recruitment (30). However, GR seems to preferably interact with GRIP1 over SRC-1, while the opposite is true for PR, which confers selectivity of GR activation and PR activation on chromatin modifications (31).

Importantly, the co-activator GRIP1 can also act as a co-repressor. Depending on the individual GR target gene, GRIP1 functions as either an activator or repressor by using its co-repressor domain. For example, GRIP1 was described to act as a co-repressor at the *osteocalcin* promoter (32). Moreover, the functionality of GRIP1 is modulated by post-translational modifications. CDK9 mediated phosphorylation of GRIP1 was shown to increase GR dependent activation, but had no effect on repression (33).

SRC-3 (NCOA3), another member of the SRC family, was originally identified through interaction with the estrogen receptor (ER) (34). Similar to SRC-1 and GRIP1, SRC-3 is recruited in a locus-specific manner (35).

In the mid-1990s, the discovery of two nuclear receptor co-repressors (NCOR)—NCOR1 (36), and NCOR2 (otherwise known as SMRT, silencing mediator co-repressor) drove further research into the field of NR co-regulators (37). The NCOR family interacts with nuclear receptors via the coRNR-box, consisting of the consensus sequence LXX I/H I XXX I/L, which contacts the AF-2 domain of NRs (38, 39). This is analogous to the LXXLL sequence in co-activators and occupies a similar location on the receptors.

While the NCOAs display intrinsic HAT activity, the co-repressors NCOR/SMRT were described to interact with the histone deacetylase HDAC3 (40). Both NCOR1 and SMRT were able to recruit HDAC3 to condense chromatin as part of their repressive mechanism (41).

SUMOylation of mouse GR at K310 was shown to be essential for repression, and in point mutant mice, neither NCOR1, SMRT nor the associated HDAC3 complex were recruited (42, 43). GCs down-regulate expression of GR itself, through a negative feedback loop. This occurs by recruitment of a GR-NCOR1-HDAC3 complex to an nGRE in exon 6 of the *NR3C1* gene (44). GC-mediated suppression of natural killer cells activity however, was described to be mediated by HDAC1 and SMRT specifically (45). The differential control of GR action by recruitment of alternative co-activators and co-repressors, in tissue or signal specific contexts, is still an open area of investigation. Different GR ligands selectively recruit alternate co-factors (46), suggesting that ligand induced conformational changes might have discrete effects on GR target genes, adding another level of complexity to GR mediated gene regulation.

Two major proteins that are recruited by co-activators are CBP (CREB binding protein) and p300. Both CBP and p300 are histone acetyl transferases (HATs), and induce chromatin relaxation (47) (Figure 2). SRC-1 was shown to recruit p300 into a complex with nuclear receptors to activate transcription (48). Part of GR's repressive action might involve competition for CBP and p300, as GR repression of an AP-1 (Activator Protein 1) reporter was abolished by overexpression of CBP and p300 (49). Moreover, enhanced engraftment of hematopoietic stem cells in response to GCs was described to be controlled by SRC-1 and p300 recruitment to the *CXCR4* gene, with acetylation of histones H4K5 and H4K16 upregulating *CXCR4* (50).

GR and the tumor suppressor protein 53 (p53) were shown to interact in a ligand dependent manner via Hd2m (a transcription factor), which enhanced the GC-induced degradation of both GR and p53 (51). In fact, the interaction between GR and p53 is important for the repression of NF-κB (nuclear factor-κB) responsive genes. Without p53, GR did not repress inflammation in a mouse model of endotoxic shock (52).

Finally, GR interacts with components of the SWI/SNF complex (SWItch/Sucrose-Non Fermentable). These highly evolutionarily conserved ATP-dependent chromatin remodelers use energy from ATP hydrolysis to alter nucleosome positioning. GR was shown to directly interact with the Baf250, Baf57, and Baf60a subunits of SWI/SNF complexes, further demonstrating the ability of GR to modify the chromatin architecture [(53–56); Figure 2].

In summary, GR recruits co-activators such as SRC family members, which in turn assemble a transcriptional complex containing histone modifying enzymes and chromatin remodelers to control the transcriptional machinery and RNA Pol II activity. These interactions are crucial for its anti-inflammatory actions and might present novel therapeutic targets in the future.

### Mechanistic Insights Into Immunomodulation From GR Point Mutations *in vitro*

Introducing point mutations into the *NR3C1* gene significantly contributed to our understanding of the molecular mechanisms of GR action. Here, we briefly address the insights gained from specific residues that revealed certain GR functions essential to suppress inflammation in cultured cells.

Besides promoter/enhancer occupancy, post-translational modifications of GR play a major role for transcriptional control. Three key phosphorylation sites were identified in the human GR: S203, S211, and S226 (57–59). All of them are located in the AF-1 domain, which is crucial for protein-protein interactions with TATA-box binding protein and others (60). By using phospho-deficient (S211A) or phospho-mimetic (S211D) mutations, it was shown that phosphorylation of GR at S211 increases association with the MED14 subunit of the mediator complex, a key bridge to the transcriptional machinery (59). In confirmation, the S211A mutant displays reduced expression of the GR targets *GILZ* and *IRF8*. S226A mutation however, had the opposite effect. The phosphorylation-deficient mutant S226A showed increased expression of *GILZ* and *IRF8*, suggesting an inhibitory role (59). In addition, S404, a site for GSK3β phosphorylation, regulates GR transcriptional activity. Mutation to S404A rewired the GR-regulated transcriptome, interestingly increasing its repressive capacity (61). Moreover, the SUMOylation-deficient murine GR K310R was shown to affect repression and the recruitment of co-regulators [(42, 43); Figure 1].

The AF-2 domain, located within the LBD (62), has additional sites modulating GR function. The mutation C656G within the AF-2 domain of the rat GR (C638 in human) reduced the ligand concentration required for activation of the *PEPCK* promoter (63). Mutations within the “charge-clamp”—that is the co-activator interaction site of K579 and E755—resulted in loss of transcriptional activation, but had no effect on repression (64).

Applying a random mutagenesis approach in yeast, Yamamoto and colleagues showed that multiple mutations within the zinc finger of the DBD impede GR binding to GREs *in vitro*, demonstrating the importance of this particular domain (65). Further mutagenesis studies in the 1990s identified a multitude of important amino acids involved in activation and repression. For example, the mutations S425G and L436V in the DBD could double the activation in a reporter assay, but almost completely abolished repression by GR (66).

Mutations in the dimer interface are also central for the understanding of GR biology. The GR^dim^ (human A458T), corresponding to rat A477T (67), and GR^mon^ (mouse A465T/I634A) (68) mutations disrupt the dimer interface. Further mutation of A458T outside the D-loop to the double N454D/A458T further increased the capacity of GR to repress a reporter *in vitro* (66). Generation of the GR(D4X), a quadruple mutant GR with the residues N454D, A458T, R460D, and D464C in the dimerization region of mouse GR provided deeper insight into the monomer/dimer action of GR. The GR (D4X) had equivalent repressive activity to wild type, while activation capacity as measured in reporter assays was near zero. This mutant demonstrated that opposition of TNF-α involved both activation of IKKB and repression, since mutant GR was unable to induce IKKB, but repressed the production of TNF-α (69). There is significant work on the GR^dim^ mutation *in vivo*, covered in the next section. Early *in vitro* work however, showed that the A477T mutation induced loss of the dimer interface and reduced DNA residence time, making target gene regulation by A477T rather difficult to interpret (70). Both wild type GR and GR^mon^ bound GRE half sites, but A447T was incapable of binding classic, full length GREs, which are occupied by receptor dimers [(67); Figure 1].

Another mutation in the second zinc finger of the DBD in rat GR R488Q (R469 in the human GR) was designed to discriminate between interactions with NF-κB and AP-1. Overexpressing GR R488Q in activated CV-1 cells under inflammatory conditions failed to suppress NF-κB reporter activity, whereas AP-1 inhibition was preserved (71). Additional GR mutations with less impact on inflammation are reviewed in more detail elsewhere (72).

Taken together, these GR point mutants show the importance and complexity of GR interactions with transcription factors and chromatin modifiers. In fact, several discrete mutations within the GR AF-1, AF-2 domains and the dimer interface alter its activity in a gene-specific manner, indicating that different parts of the receptor are dispensable for certain gene regulatory events, but essential for others (32). Differentially interfering with GR function therefore affects multiple physiological processes, and distinct types of inflammatory responses.

### Lessons Learned From Genome-Wide Studies

Chromatin as a key determinant of GR function has been highlighted in multiple genome-wide ChIP-sequencing studies since the early 2010s. For instance, GR gene regulation is determined by the chromatin architecture of the responsive cell. GR does not act as its own pioneer factor, but rather cell-type-specific gene regulation is dependent on pre-existing available binding sites, determined by chromatin accessibility (73). The pro-inflammatory transcription factor AP-1 governs a large subset of GR regulatory sites, making areas of DNA accessible to GR (74). As GR is largely dependent on pre-existing open chromatin for binding, it cemented the possibility that stimuli which are known for chromatin remodeling, for example inflammation, alters GR binding. Indeed, treatment with TNF-α amends the transcriptional response to GCs, as well as chromatin occupancy of GR, and surprisingly GR activation also transformed the occupancy of NF-κB (75). Recent data showed that GR could indeed act as a pioneer factor for other transcription factors, such as FOXA1, but only at a minority of genomic sites, and thus far this effect has not been demonstrated in immune cells (76).

When assessing GR activity in a more relevant cell-type, macrophages treated with LPS, GR, p65 (part of the NF-κB complex), and c-Jun (one of the members of the AP-1 dimer) binding overlapped significantly (see below). However, the directionality of the gene regulatory response did not correlate well with the type of interaction. That is, contrary to established models, GR binding to NF-κB loci did not only result in repression of target genes, but either repression or activation depending on the particular locus. The inverse is also true, that GR binding to canonical GREs did not only result in up-regulation of transcription at the assigned gene. Rather than the presence or absence of GR as the determining factor, the recruitment of different chromatin modifiers, such as GRIP1, were the prime measure of whether the particular gene would be activated or repressed (77).

Moreover, GR effects can be dependent on the timing of the inflammatory signal. Pre-treatment of macrophages with GCs before LPS stimulation resulted in differential gene regulation compared to treatment with GCs after LPS stimulation. In addition, a large part of GR's anti-inflammatory action can be accounted for by the induction of negative regulators of inflammation such as Mkp1, GILZ, and A20, see below (78). GR^dim^ macrophages treated with LPS and Dex also showed that the dimerization impaired GR preferentially occupied GR-half sites (16), a phenomenon also observed in cells overexpressing GR A477T (67).

Importantly, all these studies showed that GR not only binds to GREs, but occupies motifs near lineage determining factors, such as PU.1 in macrophages. Again this underscores the idea that GR requires open, pre-programmed chromatin for finding its genomic target sites (16, 74, 77–79). The chromatin landscape is cell-specific and depends on pioneer factors, cell lineage transcription factors and epigenetic marks that all predetermine GR binding. Only a minority of GR peaks are found in inaccessible chromatin and trigger chromatin remodeling upon hormone treatment (16, 73, 79–82). These findings strongly suggest that other DNA-binding proteins prime the chromatin landscape prior to GR arrival. The collaborative binding of lineage-determining transcription factors results in nucleosome remodeling, which generates open regions of chromatin. This provides access to signal-dependent transcription factors to bind open regions and modulate gene transcription in a cell-specific manner (83). In the context of macrophages, PU.1 and C/EBP are essential for the development of the myeloid lineage and have been shown to establish the monocyte-specific enhancer landscape (83, 84). PU.1 deletion results in loss of macrophages, neutrophils and B cells (85, 86). Importantly, PU.1 and C/EBP transcription factors often co-localize with GR in macrophages (16).

This new methodology has given deeper insights into the mechanisms by with GR regulates gene expression, identifying chromatin remodeling, and cooperation with other transcription factors, as a key determinants of GR activity. Importantly, GR's reliance on other factors to define its binding sites underscores the necessity of studying GC responses in a tissue-specific manner, rather than extrapolating effects from one cell-type to another.

### Molecular Mechanisms of Immunomodulation by the Glucocorticoid Receptor

#### Non-genomic Actions of GR

Some therapeutic GC effects, such as bronchodilation, resolution of airway irritation or suppression of inflammation, occur almost too rapidly to result from transcription, raising the possibility of non-genomic GR actions (87, 88). These could be GR-unspecific interactions with cellular membranes, functions of membrane-bound GR or specific interactions with cytosolic GR, thereby altering posttranslational modifications like phosphorylation, or other mechanisms (89).

Membrane-bound GR was described in human monocytes and B cells (90, 91), and non-genomic functions have been found in macrophages (92), lung epithelial cells (93), and T-cells (94).

Downstream of inflammatory MAPK signaling, mitogen- and stress-activated protein kinase-1 (MSK1) is an essential kinase for NF-κB p65 S275 phosphorylation (95).Interestingly, GC-mediated repression of NF-κB targets involves loss of MSK1 kinase recruitment at inflammatory promoters and nuclear export of MSK1 via cytosolic GR (96). Putatively, GR can also crosstalk with AKT, GSK-3β, and mTOR signaling (93).

These non-genomic effects might be very interesting for the development of novel therapeutics, and will benefit from future studies, for example with novel cell lines or mouse models to dissect these complex interactions.

#### Genomic Actions of GR

**Figure 3:**
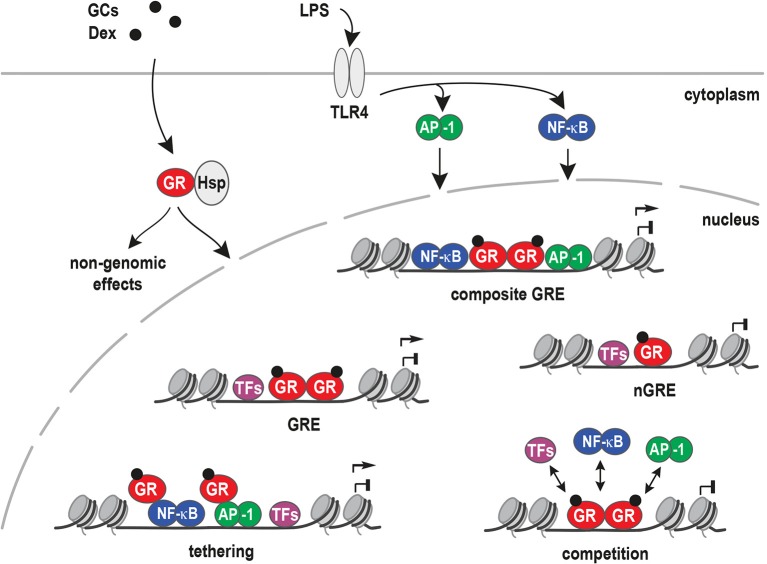
Models for inflammatory gene regulation by the glucocorticoid receptor. Upon ligand binding (GCs), the glucocorticoid receptor (GR) is released from heat shock proteins (Hsp) and translocates to the nucleus. Inflammation can be activated by lipopolysaccharide (LPS) binding to Toll-like receptor 4 (TLR4). TLR4 signaling results in the activation of NF-κB, AP-1, and other inflammatory transcription factors that bind and regulate pro-inflammatory target genes. Different mechanisms have been proposed for GR's potent anti-inflammatory actions, i.e., binding to Glucocorticoid Response Elements (GREs), to composite GREs together with other transcription factors, to negative GREs (nGRE), by tethering to DNA-bound transcription factors, by competing with other factors for DNA binding sites or by non-genomic actions.

Lipopolysaccharide (LPS) is a molecular component of the cell wall of Gram-negative bacteria commonly used to study inflammation (97, 98). On macrophages, LPS binds to Toll-Like Receptor 4 (TLR4) and activates a signaling cascade that results in NF-κB and AP-1 nuclear translocation. Together with other inflammatory transcription factors, these two protein complexes then activate pro-inflammatory gene expression (99, 100). TLR4 activates AP-1 via the MAPK signaling pathway and NF-κB via degradation of the cytosolic IKK complex that frees the NF-κB transcription factor (Figure 3).

GR can antagonize or synergize with pro-inflammatory signaling, depending on the context of promoters or enhancers. For antagonism of pro-inflammatory signaling, several mechanisms are proposed. These include the direct interference with MAPK or JNK signaling (101, 102), leading to repressive actions at the gene regulatory level. Conversely, repression of GR-target genes might be explained by tethering to other transcription factors or trans-repression, negative GREs (nGREs, with a different sequence), composite GREs, non-canonical novel GREs, DNA as a modulator of GR, and consensus classical GREs.

Most frequently, GR tethering to AP-1 or NF-κB via protein-protein interactions (trans-repression), instead of direct DNA binding, was suggested to underlie its repression of inflammatory responses (103, 104). In other words, GR has been shown to represses genes via protein-protein interactions with AP-1 (105), NF-κB (106), STAT3 (107), and other DNA-bound transcription factors (Figure 3). Interestingly, STAT3 tethering to GR resulted in synergistic gene regulation, and increased target gene expression in AtT-20 cells. On the other hand, GR tethering to DNA-bound STAT3 resulted in transcriptional repression (107).

Negative GREs (nGREs) were originally described as GREs motifs in the promoters of repressed target genes. nGREs can be found in very different cell types and genes involved in various processes, for example: HPA axis (*POMC* and *CRH*) (108, 109), lactation (*PRL3*) (110, 111), bone homeostasis (*osteocalcin*) (112), skin structure (*keratins*) (113), and inflammation (*IL-1*β) (114).

However, the definition of nGREs has not yet reached consensus in the literature, and subsequently, GBS with non-classical consensus sequences, near repressed targets, are also named nGREs. One study described a variation of nGREs, termed “inverted repeat (IR) nGRE.” IR nGRE is a complex GBS with the following consensus motif: CTCC(n)_0−2_GGAGA, which differs from the classical GRE (AGAACAnnnTGTTCT) or nGRE (115). These elements however, have not been identified by ChIP-seq, questioning how relevant they are to GR responses.

Similar to nGREs, composite elements, such as degenerate GREs overlapping with other transcription factor consensus motifs, may also affect the transcription of inflammatory targets. For example, a 25-base pair composite element (plfG element) in the promoter of the *proliferin* gene, is regulated by GR and AP-1 (116, 117). Furthermore, the GR DNA-binding domain (DBD) can bind a newly identified motif inside NF-κB consensus sequences. Crystal structures of the GR DBD demonstrated direct binding of GR to the AATTT nucleotides within the NF-κB motif from the promoter regions of *CCL2, IL-8, PLAU, RELB*, and *ICAM1*. This cryptic GR-binding site overlapping the NF-κB response element was named κBRE and was highly conserved between species (118).

An important aspect is the concept of DNA being an allosteric modulator of the GR. Here, the precise nucleotide sequence in a GBS is proposed to function as a shaping ligand that specifies GR's transcriptional activity. X-ray crystallography of GR DBD dimers bound to different GBSs showed that conformation of the lever arm in the DBD appeared to be influenced by the DNA sequence (24, 119). Furthermore, the addition of a single GR-binding site was sufficient to convert a gene, which was normally not regulated by GR, into a target gene, such as *IL-1*β and *IL1R2* in U2OS cells (120). The presence of classical GREs in GR-bound enhancers near both activated and repressed genes in murine bone marrow-derived macrophages (BMDM) stimulated with LPS and Dexamethasone (Dex) challenge these models. These findings suggest that first, direct GR:GRE binding is relevant for repression of inflammatory genes. Secondly, that the classical models described above are not sufficient for prediction of GR mediated activation or repression. Therefore, the presence of a different combination of cofactors in activated vs. repressed sites could explain or contribute to the up- or down-regulation of GR target genes (77, 118, 121, 122).

Taken together, how GR activates one set of target genes while repressing another is still an open question, and the molecular mechanisms specifying the repression of inflammatory genes remain unknown. Repression by GR is a complex process which likely involves different determinant factors. One factor is GR itself (phosphorylation, post-translational modifications and ligand-specific conformations), another factor is the DNA sequence, the cell type-specific chromatin landscape and the cooperation with co-regulators and other transcription factors. All of these, together with potentially unknown factors, ultimately determine which target genes are up- or down-regulated.

### Mechanistic Insights Into Immunomodulation From GR Point Mutations *in vivo*

As described above, one particular class of point mutations, which interfere with GR dimerization, caught considerable attention. In tissue culture experiments expressing these GR^dim^ mutants (human GR A458T, mouse GR A465T, and rat A477T), the concept was developed that abrogation of dimerization could be beneficial to limit side effects of anti-inflammatory treatments. Therefore, pharmaceutical companies directed their research to develop dissociated ligands favoring GR monomer dependent favorable effects and reducing unwanted GR dimer action (123, 124).

Various selective GR agonists (SEGRAs), such as RU24858, RU24782, and non-steroidal ligands (LDG552, ZK216348, Compound A), were examined for desired anti-inflammatory effects with the hope that there would be minimal metabolic actions (124, 125). Only a few of these compounds, however, showed promise in preclinical trials (126). Their limited success arose from the generalized and oversimplified view that the GR monomer mediates trans-repression (anti-inflammatory) and the GR dimer regulates only unwanted effects (127). The disappointing conclusion of these programs for SEGRAs and non-steroidal ligands and their translation to the clinic called for new perspectives in the context of pathophysiology (10, 16, 104, 127–129). With knowledge gained from the GR^dim^ mouse and others, the development of selective monomerizing GRagonists or modulators (SEMOGRAMs) and selective dimerizing GRagonists or modulators (SEDIGRAMs) has begun to make progress (130). To find SEDIGRAMs, a screening identified Cortivazol and AZD2906 as compounds that increase GR dimerization and enhance the transactivation capacity. Both chemicals, however, still have GR monomer activity, indicating that these are not yet the ideal SEDIGRAMS (129). Efforts are still ongoing to identify perfect GR modulators separating dimer from monomer.

**Figure 4:**
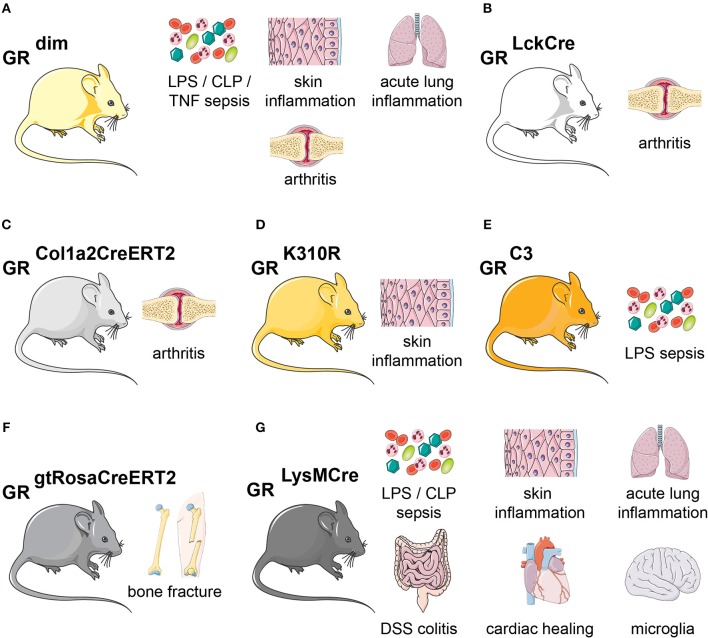
Glucocorticoid receptor mutant mouse models of inflammation. Overview of the mouse lines discussed in this article. **(A)** GR^dim^ mice are more sensitive during LPS-, CLP-, or TNF inflammation. GR^dim^ mice are refractory to GC treatment in models of skin inflammation, acute lung injury and arthritis. **(B)** In GR^LckCre^ mice, GR is lacking in T-cells, making them refractory to GC treatment during arthritis. **(C)** GR^Col1a2CreERT2^ (lacking GR in fibroblasts) show delayed GC-induced suppression in arthritis. **(D)** GR K310R mutant mice lack GR SUMOylation and show impaired control of skin inflammation. **(E)** GR-C3 mice, lacking the most active GR isoform C3, are more sensitive to LPS-induced endotoxic shock. **(F)** During fracture, GR is necessary in all cells, as shown by GR^gtRosaCreERT2^ (tamoxifen-induced ubiquitous Cre-mediated recombination) for fracture healing. **(G)** GR^LysMCre^ mice (GR is deleted in myeloid cells) show no proper healing in LPS-or CLP-sepsis, skin inflammation, acute lung injury, DSS colitis, cardiac healing, and Parkinson disease. The skin, lungs, bones, intestine, heart and brain cartoons were obtained from Servier Medical Art.

In 1998, the GR A465T mutation was introduced into mice (131, 132). Intriguingly, mice born with this mutation survived in certain backgrounds (131), and simple inflammatory models, such as phorbol ester induced skin irritation, responded to GC treatment in these animals. This indicated that GR monomer and thus transrepression by tethering might be sufficient to reduce inflammation. However, for most other inflammatory models, GCs failed to have an effect in these GR^dim^ mice (Figure 4A).

For instance, during LPS, CLP (cecal ligation and puncture), and TNF-α induced shock, GR^dim^ mice were highly susceptible to inflammation and cytokine production, impaired thermoregulation and metabolic alterations (133–135). Furthermore, macrophages from GR^dim^ mice were unable to efficiently repress cytokines in response to LPS (135). Moreover, GR^dim^ mice treated with exogenous GCs showed impairment of anti-inflammatory responses in models of acute lung injury (ALI), arthritis, contact allergy, and allergic airway inflammation (136–139). During ALI, this was partially due to diminished expression of the GR-dimer target gene *Sphk1* (138) (see above). In models of allergic airway inflammation, contact hypersensitivity, antigen-induced arthritis (AIA) or serum transfer-induced arthritis (STIA), GR^dim^ mice failed to repress inflammation when given GC therapy (136, 137, 139, 140). In the model of AIA, GR dimerization was shown to be essential in T cells (GR^LckCre^ mice) to reduce inflammation [(137); Figures 4A,B]. More recently, GR^dim^ mice reconstituted with wild type hematopoietic stem cells failed to induce non-classical (CD11b^+^, F4/80^+^, Ly6C^−^), non-activated (CD11b^+^, F4/80^+^ MHCII^−^), anti-inflammatory (CD163, CD36, AnxA1, Axl, and MertK) macrophages during STIA, while cytokines were repressed normally (140). This strongly indicated that intact dimerization in stromal non-immune cells could contribute to the suppression of inflammation. More precise, the GR in fibroblast-like synoviocytes (GR^Col1a2CreERT2^) was crucial to reduce STIA (140) (Figure 4C). GR^dim^ mice were also resistant to GC treatment during TNF-induced inflammation, and exhibited increased gut barrier leakiness, cell death of intestinal epithelial cells and cell death. An increased STAT1-responsive interferon-stimulated gene signature was observed in the gut of GR^dim^ mice (141).

Whereas, the GR^dim^ knock-in mice were intensively studied, less is known about other point mutations. The GRK310R mutation, which abrogates SUMOylation of the GR, failed to respond to GCs during skin inflammation. This was in part due to reduced SMRT/NCoR-co-repressor recruitment to GR/NF-κB/AP-1 repressive complexes [(42, 43); Figure 4D].

Finally, Cidlowski and colleagues published a knock-in mouse of the most active GR isoform C3. The lethality of these mice could be overcome by antenatal GC administration, and adult mice were hypersensitive to LPS administration. This indicated that either the absence of other isoforms like the most abundant GR-A, or indeed the specific overexpression of GR-C3 might confer anti-inflammatory actions [(142); Figure 4E]. However, further studies are warranted to dissect these observations in more detail.

Taken together, GR point mutations introduced *in vivo*, namely the GR^dim^ mutation, but also the more recent mutations, have yield valuable insight into the molecular features of GR. With the emergence of CRISPR/Cas9 gene editing technology, more *in vivo* models for specific GR functions will help our understanding of GR in physiological processes in the future.

### Glucocorticoid Action on Macrophages

GCs exert their immunosuppressive effects through many cells of the innate immune system, including dendritic cells, mast cells, neutrophils, and eosinophils (143, 144). GCs also play a major role in the regulation of adaptive immunity. For example, GCs decrease the proliferation of early B cell progenitors (145) and induce apoptosis in B cells and T cells (145–149). In this review, we will focus mainly on the effects of GCs in macrophages, since these innate immune cells are essential mediators of defense responses, beyond the mere removal of pathogens, and regulate tissue homeostasis in a myriad of ways (150).

Macrophages reside in many different tissues and are the first line of defense against pathogens (151). Depending on the activating stimulus, they can be categorized as M1-like and M2-like macrophages. The M1-like macrophages (classically activated macrophages) mediate pro-inflammatory actions. They are activated by exposure to LPS, INFγ, TNF-α, or pathogen- and danger-associated molecular patterns (PAMPs and DAMPs, respectively) (151–153). GCs suppress inflammatory responses downstream of TLRs, in part by interfering with the NF-κB- and AP-1-activated transcription of pro-inflammatory cytokines and chemokines (154, 155).

The M2-like macrophages on the other hand, are characterized by their anti-inflammatory potential and are activated by cytokines involved in inflammatory resolution, like IL-4, IL-10, and IL-13 (151, 153, 156). GCs can also polarize macrophages to an M2-like phenotype by regulating the expression of anti-inflammatory proteins (153, 156). A major, yet undervalued aspect of GC control of anti-inflammatory macrophage polarization is the regulation of efferocytosis. GCs enhance the clearance of apoptotic cells, which in itself can augment the development of an anti-inflammatory macrophage phenotype (157, 158).

In sum, GCs can modulate macrophage activity in a number of different and intricate ways, which include suppressing the production of pro-inflammatory proteins and inducing anti-inflammatory mediators.

#### Glucocorticoid Receptor Target Genes Mediating Immune Modulation

GC stimulated macrophages shift to an M2-like anti-inflammatory and inflammation-resolving phenotype (156). These effects are achieved by the repression of pro-inflammatory genes, the induction of gene products antagonizing pro-inflammatory signaling, and by synergism with pro-inflammatory signaling pathways to activate genes resolving inflammation.

While the mechanisms of gene repression have been extensively discussed [referring to interleukins, chemokines, matrix metalloproteinases, inducible nitric oxide synthase (iNOS), and other mediators], the activated anti-inflammatory genes have only recently received attention (Table 1).

**Table 1:** GR target genes relevant for (anti-) inflammatory action.

**GC-regulated genes**	**Targets**	**GC effect on immune responses**	**References**
Cytokines	Il-1α, Il-1β, Il-6, Il-8, and Il-12	Repression of cytokine production	(114, 159, 160)
Chemokines	Ccl2, Ccl3, Ccl4, Cxcl9, and Cxcl11	Suppression of chemokine release	(77, 160–162)
Matrix metalloproteinases	Mmp12 and Mmp13	Reduction of extracellular matrix remodeling, proteolytic processing	(77, 161)
MAPK phosphatase 1	Induction of Mkp1	Suppression of Jnk and p38Mapk	(133, 163–169)
GC-induced leucine zipper (Tscd22d3)	Induction of Gilz	Inhibition of NF-κB	(170–177)
IκBα and IκBβ	Induction of IκBα and IκBβ	Trapping NF-κB in the cytoplasm, reduced NF-κB activity	(178, 179)
Kruppel-like factor 2	Induction of Klf2	Competition with AP-1 and NF-κB, reduction of inflammatory cytokines	(180–182)
Kruppel-like factor 4	Induction of Klf4	Inhibition of NF-κB	(180, 183)
A3 adenosine receptor	Upregulation of A3AR	Enhanced Erk1/2, anti-apoptotic and pro-survival	(184)
Annexin A1	Induction of Annexin A1	Induction of efferocytosis and monocyte recruitment	(185–189)
Pparγ	Upregulation of Pparγ	Reduced migration	(190)
Tristetraprolin	Induction of TTP	Destabilization of TNF-α	(191–193)
Irak-M	Irak-M induction through synergistic action of GC/GR and NF-κB	Suppression of pro-inflammatory mediators	(193, 194)
Sphingosine Kinase 1	Sphk1 induction through synergism of GC/GR and p38Mapk-Msk1	Reduced vascular leakage and infiltration during acute lung injury	(138)
Serpin A3	Serpin A3 induction through synergism GC/GR and TNFSR1	GR recruitment to Serpin A3 TSS by Dex and TNF-α treatment	(195)
Metallothioneins	Mt1 induction through synergism of Il-6 and GC/GR	Increased susceptibility in inflammatory model in the absence of Mts	(196–202)

Prominent examples are the induction of MAPK phosphatase 1 (Mkp1 or Dusp1), that interferes with the p38MAPK pathway; GC induced leucine zipper (GILZ/Tsc22d3), which binds to the NF-κB subunit p65; the induction of IκBα and β, which oppose NF-κB activity; the activation of kruppel like transcription factors (Klf), which are important for alternative macrophage polarization, and many others (Table 1). This upregulation of anti-inflammatory genes further emphasizes that both gene repression and activation are required for the immunomodulatory effects of GCs.

More recently, there were intriguing observations that GCs not only antagonize inflammatory signaling, but also synergize with pro-inflammatory signaling pathways (Table 1). GCs synergize with *Haemophilus influenzae* activated inflammatory pathways in macrophages, bronchial epithelial cells (BEAS-2B) and lung epithelial cells (A549) to induce IRAK-M, a negative regulator of TLR signaling (203). Mechanistically, this synergistic activation of *Irak-M/Irak-3* transcription is dependent on binding of both GR and p65 to its promoter, showing a cooperative induction by NF-κB and GR that limits inflammation (203). Similarly, GCs activate TLR2 expression synergistically with *H. influenza* signaling *in vitro* (194).

In ALI models, GR was shown to cooperate with LPS-induced p38MAPK-Msk1 to induce Sphingosine Kinase 1 (SphK1) expression in macrophages (138). SphK1 produces the active mediator Sphingosine-1-phosphate (S1P), that binds to the S1P receptor 1 (S1PR1) on endothelial cells to reduce vascular leakage and infiltration during lung inflammation (138, 204–208). In ALI, mice lacking SphK1 in macrophages were resistant to GC treatment and showed reduced S1P levels. Additional examples of synergistically regulated genes important for modulation of inflammation are acute phase proteins like Serpin A3 (α1-antichymotrypsin) (195) and Metallothioneins (Mt1 and Mt2) (196, 197).

The synergistic regulation of immune-modulating genes by GCs and pro-inflammatory pathways is an important component of their mechanism, but the underlying dynamics and time windows are still poorly understood.

#### Loss of Function Models of GC Signaling in Macrophages

Strong evidence for the role of GR during homeostasis and inflammation was derived from conditional loss-of-function studies in mice. Applying the Cre/LoxP system, GR tamoxifen-inducible mice (GR^gtROSACreERT2^) could be used to determine the impact of GR deletion in adult animals, circumventing the lethality of global GR knockouts. For example, they have been useful to study GR during inflammation-dependent bone repair after fracture (209). Overall, the mice displayed a mild increase in inflammation, with elevated serum IL-6 levels and increased IL-1β levels at the fracture hematoma, accompanied by increased CD3^+^ and CD8^+^ cells. Consequently, the lack of GR and potentially the elevated inflammation, caused a delayed endochondral regeneration and maturation of callus and a decreased healing response [(209); Figure 4F].

Since the publications of conditional GR alleles in 1999 (210), 2003 (211), and 2012 (212), many cell types have been targeted with specific Cre lines to characterize specific functions of the GR in numerous cell types in the brain, muscle, heart, T lymphocytes, and others.

Insights into the function of GR in macrophages *in vivo* mainly stems from Lysozyme 2 (LysM)–Cre mice crossed to GR floxed alleles, which causes deletion in the myeloid cell lineage (monocytes, mature macrophages, and granulocytes) [(135, 136, 163, 213); Figure 4G].

In both the LPS-induced endotoxic shock model and during CLP, myeloid GR is crucial for the repression of inflammatory cytokines and for survival (135, 163). Not only in LPS-induced inflammation, but also in dextran sodium sulfate (DSS)-induced colitis, the action of endogenous GCs in macrophages was essential to reduce intestinal inflammation (214). Mice deficient for macrophage GR had a higher disease score, with increased infiltration of neutrophils, T cells and macrophages in the colon, which was associated with enhanced serum IL-6 (214). Moreover, macrophages were shown to play an essential role for cardiac healing, tissue repair and hence survival in myocardial infarction (215). Deletion of GR in macrophages delayed cardiac healing 7 days after myocardial infarct, with impaired cardiac function, collagen scar formation and neovascularization, and larger myofibroblasts. Consequently, targeting macrophage GR during myocardial infarction might be a potential pharmacological intervention for tissue repair (215). In contrast, in a mouse model of atherosclerosis, macrophage GR deletion was beneficial and showed reduced levels of vascular calcification, due to reduced RANKL, BMP2, and Mx2 expression (216).

During skin inflammation in a model of contact hypersensitivity, the anti-inflammatory effects of GCs required GR in myeloid cells (136). Additionally, in a model of ALI, GR^LysMCre^ mice were resistant to GC therapy, did not reduce cellular infiltration in the lung and did not induce the endothelial barrier stabilizing sphingosine-1-phosphate [(138); Figure 4G].

GR^LysMCre^ mice were shown to efficiently express Cre in microglia, knocking out GR in brain resident macrophages. Studies on the function of microglial GR during acute inflammation demonstrated more cellular lesions, damage, demyelination in the corpus callosum, and increased neuronal degeneration. It also significantly increased pro-inflammatory cytokines after LPS injections (217). The activation of microglia induces secretion of pro-inflammatory proteins that contribute to dopaminergic neuronal death, a major a hallmark of Parkinson's disease. The absence of GR in microglia revealed that increased death of dopaminergic neurons in Parkinson's may contribute to neurodegenerative processes (218). Additionally, recent studies suggest that the absence of microglia GR facilitates TLR9 activation of inflammatory processes and affects Parkinson's disease progression (219).

In summary, the genetic deletion of GR in myeloid cells in various inflammatory models demonstrated the pivotal role of this cell type for GC actions. However, one of the limitations of the LysMCre mouse is the recombination in other myeloid cells such as neutrophils, whose contribution cannot be excluded. Nonetheless, this wealth of data supports the concept that selective targeting of glucocorticoids to macrophages, while sparing other cell types, could be a promising approach to optimize therapy.

## Conclusion

During the past decade, much has been learned about the immunomodulatory mechanisms employed by GR: analyzing various mouse models, creating distinct mutations, mapping GR target genes genome-wide, functionally characterizing individual proteins mediating GC responses, studying different inflammatory settings, identifying essential co-regulators, and applying novel molecular biology methods, have broadened our understanding of these steroids' intricate actions. Taken together, it becomes obvious how basic research is fundamental in enabling drug development. However, we now realize that GR's molecular mechanisms are very complex, cell-type, locus- and signal-specific, and much more sophisticated than we previously anticipated. Intra- and extra-cellular signals can control GR function on many levels, and these multi-layered machineries demand new interpretation of previous over-simplified models. In the future, the rapid advancement of high-throughput technologies such as machine learning, genomics, proteomics, genome engineering, etc. will be key to the development of safer immunomodulators or novel GR ligands.

## Author Contributions

LE-T, GC, AM, and SV wrote the manuscript with supervision of JT and NU. SV, JT, and NU secured funding.

### Conflict of Interest Statement

The authors declare that the research was conducted in the absence of any commercial or financial relationships that could be construed as a potential conflict of interest. The handling Editor declared a past co-authorship with one of the authors JT.
